# Synthesis, structure, magnetic and half-metallic properties of Co_2−*x*_Ru_*x*_MnSi (*x* = 0, 0.25, 0.5, 0.75, 1) compounds

**DOI:** 10.1107/S2052252519015641

**Published:** 2020-01-01

**Authors:** H. P. Zhang, W. B. Liu, X. F. Dai, X. M. Zhang, H. Y. Liu, X. Yu, G. D. Liu

**Affiliations:** a State Key Laboratory of Reliability and Intelligence of Electrical Equipment, Hebei University of Technology, Tianjin 300130, People’s Republic of China; b School of Materials Science and Engineering, Hebei University of Technology, Tianjin 300130, People’s Republic of China

**Keywords:** half-metals, electrical transport, structural properties

## Abstract

A series of Co_2−*x*_Ru_*x*_MnSi (*x* = 0, 0.25, 0.5, 0.75, 1) Heusler compounds were successfully synthesized. The heat-treatment conditions were crucial to make the materials form a single phase with a Heusler structure. With increasing Ru content, the half-metallic gap, lattice parameters and magnetization are continuously adjustable in a wide range. The Co_2−*x*_Ru_*x*_MnSi (*x* = 0, 0.25) compounds are rigorous half-metals and show a *T* ^3^ dependence of resistance at low temperature. The Co_2−*x*_Ru_*x*_MnSi (*x* = 0.5, 0.75, 1) Heusler compounds are the nearly half-metallic materials and show a semiconductive dependence of resistance at low temperature. The experimental magnetization is consistent with that in theory and follows the Slater–Pauling rule. The Curie temperature is higher than 750 K for all Co_2−*x*_Ru_*x*_MnSi Heusler compounds.

## Abstract 2

The structural, electronic, magnetic and transport properties of half metallic Co_2−*x*_Ru_*x*_MnSi (*x* = 0–1) Heusler compounds are studied both theoretically and experimentally. Our computations and experiments soundly agree with each other.

## 1. Introduction   

Half-metals, a class of materials with 100% spin polarization of conduction electrons, have attracted considerable attention owing to their potential applications in spintronic devices. Since De Groot *et al.* first reported that the NiMnSb Heusler compound has half-metallic (HM) characteristics, Heusler compounds have become a focused field to develop materials with a high spin-polarized ratio of conduction electrons (De Groot *et al.*, 1983 ▸). In the last two decades, a large number of theoretical searches for half-metals in Heusler compounds have been carried out based on first-principles calculations (Rached *et al.*, 2015 ▸; Zenasni *et al.*, 2013 ▸; Dahmane *et al.*, 2016 ▸; Wei *et al.*, 2017 ▸; Zhang *et al.*, 2019 ▸; Picozzi *et al.*, 2002 ▸; Wang *et al.*, 2017 ▸). According to these theoretical results, many experiments to synthesize Heusler compounds have also been performed (Taşkın *et al.*, 2017 ▸; Zhao *et al.*, 2019 ▸; Alzyadi *et al.*, 2016 ▸; Umetsu *et al.*, 2004 ▸; Dai *et al.*, 2009 ▸; Du *et al.*, 2013 ▸). In particular, more attention has been paid to Heusler compounds with stoichiometric composition. Basic physical properties, such as magnetic, electronic and transport properties were also investigated for many Heusler compounds with half-metallicity or nearly half-metallicity (Raphael *et al.*, 2001 ▸; Bombor *et al.*, 2013 ▸; Rani *et al.*, 2018 ▸; Prestigiacomo *et al.*, 2014 ▸). Although a large number of theoretical and experimental works have been carried out, the HM material systems with high Curie temperatures, continuously adjustable magnetism, flexible lattice parameters and different HM band gaps are still highly desired at present.

Co_2_-based Heusler compounds are very promising for spintronics applications, including giant magnetoresistance devices (Du *et al.*, 2015 ▸), magnetic tunnel junctions (Hu *et al.*, 2016 ▸) and spin injection into semiconductors (Uemura *et al.*, 2015 ▸), owing to their high Curie temperatures, high spin polarization and high magnetization. As we know, the Co_2_MnSi Heusler compound is a good half-metal with a Curie temperature higher than 985 K and a total magnetic moment of about 5 μ_B_ per unit cell (Webster, 1971 ▸). Hu *et al.* experimentally investigated the temperature dependence of spin-dependent tunneling conductance of magnetic tunnel junctions with HM Co_2_MnSi electrodes (Hu *et al.*, 2016 ▸). Some investigations on the doping effect have also been performed for the Co_2_MnSi Heusler compound (Rani *et al.*, 2018 ▸; Prestigiacomo *et al.*, 2014 ▸; Umetsu *et al.*, 2008 ▸; Kubota *et al.*, 2009 ▸; Jia *et al.*, 2008 ▸; Guezlane *et al.*, 2016 ▸;). For instance, the magnetic, structural and transport properties of Co_2_MnSi_1−*x*_Al_*x*_ compounds have been investigated (Umetsu *et al.*, 2008 ▸; Kubota *et al.*, 2009 ▸; Jia *et al.*, 2008 ▸). Jia *et al.* have predicted that Co_2_MnSi_1−*x*_Al_*x*_ compounds have spin-polarized charge carriers and carrier-dependent magnetotransport measurements should provide interesting electrical conduction properties when the Fermi level is systematically shifted through majority/minority spin bands by doping (Jia *et al.*, 2008 ▸).

One of the most attractive features of Heusler compounds is the possibility to continuously tune their saturation magnetization (*M*_t_), Curie temperature (*T*_C_) and Fermi level position by changing the number of valence electrons (*Z*_t_). In this paper, we discuss our detailed theoretical and experimental studies on the effect of Ru-doping to the structural, electronic, magnetic and transport properties of the Co_2_MnSi Heusler compound. Firstly, the Co_2−*x*_Ru_*x*_MnSi (*x* = 0, 0.25, 0.5, 0.75, 1) Heusler compounds have been successfully synthesized by a proper heat-treatment condition and HM gap; secondly, the lattice parameters and magnetization are continuously adjustable over a wide range with increasing Ru content; thirdly, the compounds exhibit a real half-metallicity when *x* < 0.7 and nearly half-metallicity when *x* ≥ 0.7.

## 2. Computational and experimental methods   

The density of states, band structures and magnetic moments were calculated by the *Cambridge Serial Total Energy Package* (*CASTEP*) code where the pseudo-potential plane-wave method was implemented (Segall *et al.*, 2002 ▸). The interactions between the atomic core and the valence electrons were described by the ultra-soft pseudo-potential approach. The generalized gradient approximation (GGA) was adopted for the exchange-correlation potential (Perdew *et al.*, 1992 ▸). A plane-wave energy cutoff of 500 eV was used for all of the calculations. A k-point mesh of 12 × 12 × 12 was used for Brillouin zone integration. The calculations continued until the energy deviation was less than 1 × 10^−6^ eV per atom.

The polycrystalline ingots of Co_2−*x*_Ru_*x*_MnSi (*x* = 0, 0.25, 0.5, 0.75, 1) were prepared by arc melting pure metals (≥99.99%) under an argon atmosphere. The ingots were melted four times to ensure chemical homogeneity. Then, the ingots were sealed in a quartz tube under high vacuum and annealed at 1173 K for 48 h. The ingots were subsequently cooled down to room temperature in the furnace. We analyzed the structures of all the samples using powder X-ray diffraction (PXRD) with Cu *K*α radiation at room temperature. Magnetization and thermo-magnetic curves were obtained using a vibrating sample magnetometer attached to the physical property measurement system (PPMS). We measured the magnetization in fields up to 5 T at 2 K and the thermo-magnetic curves under a field of 1000 Oe. The resistance measurements were performed using the four-probe method in PPMS.

## 3. Results and discussions   

### 3.1. Electronic structures and half-metallicity   

**Figure 1:**
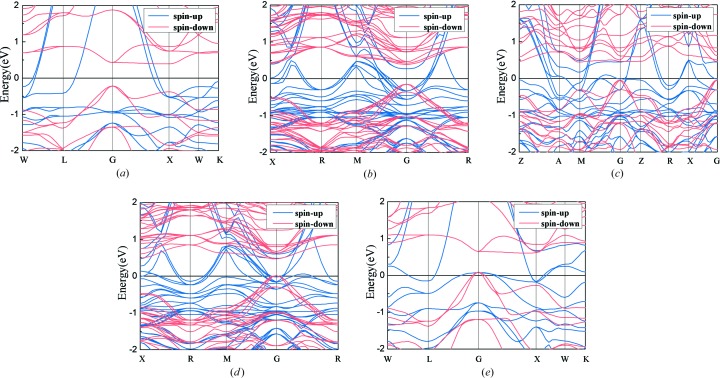
Calculated band structures of Co_2−*x*_Ru_*x*_MnSi alloys for (*a*) *x* = 0, (*b*) *x* = 0.25, (*c*) *x* = 0.5, (*d*) *x* = 0.75 and (*e*) *x* = 1 at their optimized lattice parameters.

The equilibrium lattice parameters (ELPs) in theory were achieved by geometry optimization to minimize the total energy and are gathered in Table 1 ▸ for the Co_2−*x*_Ru_*x*_MnSi (*x* = 0, 0.25, 0.5, 0.75, 1) Heusler compounds. All of the following theoretical calculations were performed based on the ELP. Fig. 1 ▸ shows the band structures of the Co_2−*x*_Ru_*x*_MnSi (*x* = 0, 0.25, 0.5, 0.75, 1) compounds. It is clear that for all the Co_2−*x*_Ru_*x*_MnSi (*x* = 0, 0.25, 0.5, 0.75, 1) compounds, the Fermi level has a metallic intersection with some of the bands in the spin-up channel. This indicates that the spin-up electrons have a strong metallic characteristic in these compounds. However, in the spin-down band structures, we can see that there exists a band gap near the Fermi level for all the Co_2−*x*_Ru_*x*_MnSi (*x* = 0, 0.25, 0.5, 0.75, 1) compounds. For further detailed observation, it can be found that Fermi level lies in the band gap for the Co_2−*x*_Ru_*x*_MnSi compounds with *x* = 0, 0.25, 0.5, indicating that these compounds are rigorous HM materials. For the cases where *x* = 0.75 and *x* = 1, their Fermi level slightly crosses through the top of the valence bands at the G point in the spin-down channel. That is to say, CoRuMnSi and Co_1.25_Ru_0.75_MnSi are not rigorous HM materials and are usually referred to as nearly HM materials (Continenza *et al.*, 2001 ▸). Nearly HM materials can be generated by the 100% spin-polarization of one of two kinds of carriers participating in electrical conduction.

**Figure 2:**
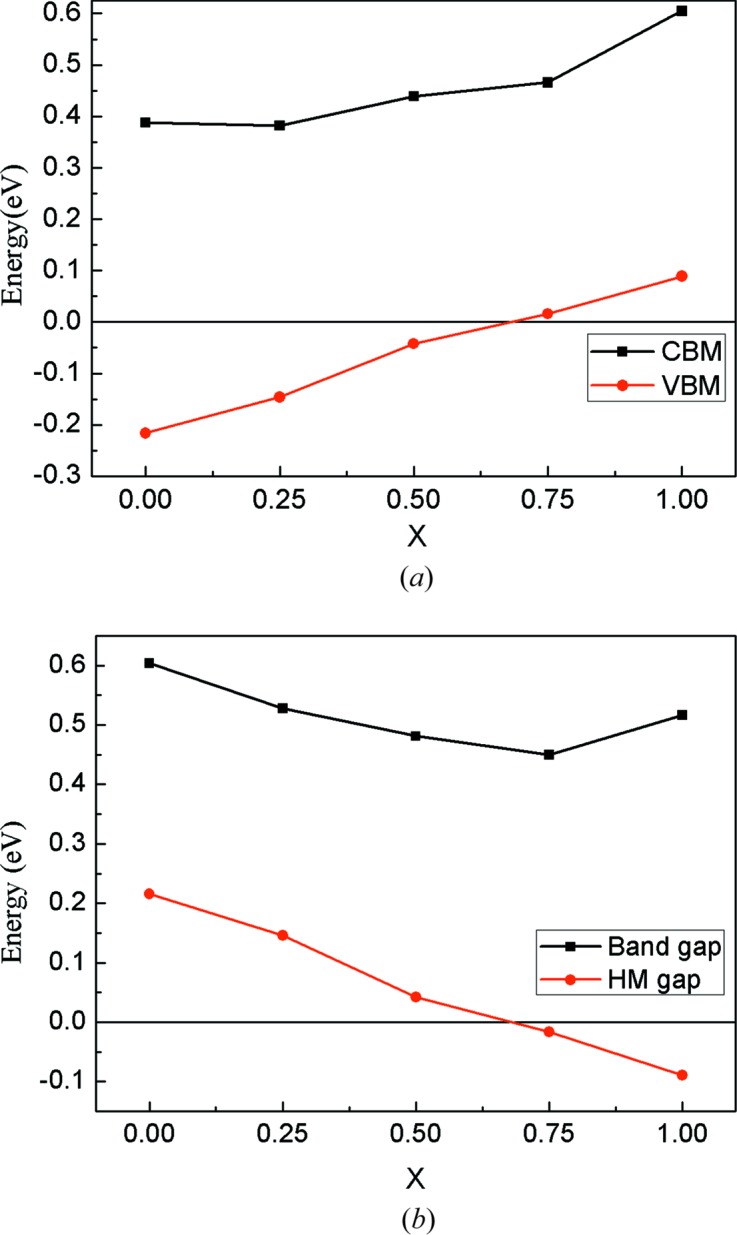
(*a*) CBMs and VBMs in the minority spin channel as functions of the Co_2−*x*_Ru_*x*_MnSi (*x* = 0, 0.25, 0.5, 0.75, 1) alloys. (*b*) Calculated band gap and HM band gap as functions of the Co_2−*x*_Ru_*x*_MnSi (*x* = 0, 0.25, 0.5, 0.75, 1) alloys.

**Figure 3:**
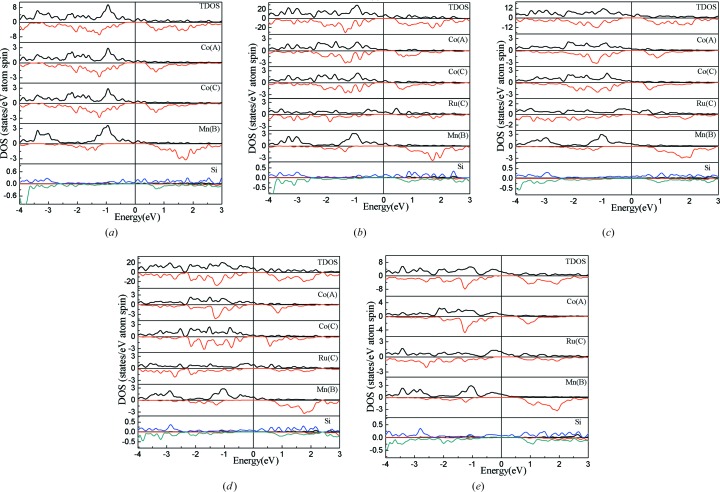
Calculated TDOS and PDOS of Co_2−*x*_Ru_*x*_MnSi alloys for (*a*) *x* = 0, (*b*) *x* = 0.25, (*c*) *x* = 0.5, (*d*) *x* = 0.75 and (*e*) *x* = 1 at their optimized lattice parameters.

It is known that the band gap and the spin-flip band gap in the spin-down channel are very important to achieve a stable high spin polarization of carriers in practical applications. The spin-flip band gap refers to the smaller of the conduction band minimums (CBM) and the absolute value of the valence band maximum (VBM) (Guo *et al.*, 2018 ▸). The larger both the gap and the spin-flip band gap are, the more stable the high spin polarization will be. Fig. 2 ▸(*a*) shows the CBM and VBM dependence on Ru content for the Co_2−*x*_Ru_*x*_MnSi compounds. In Fig. 2 ▸(*a*), it can be observed that the CBM monotonically increases with increasing Ru content, and VBM decreases with increasing Ru content up to *x* = 0.7, where the VBM becomes positive and the half-metallicity disappears. Fig. 2 ▸(*b*) shows the band gap and the spin-flip band gap dependence on the Ru content for the Co_2−*x*_Ru_*x*_MnSi compounds. The band gap is larger than 0.45 eV and varies only slightly with the change in Ru content. And the spin-flip band gap decreases with increasing Ru content because the Fermi level moves towards lower-value bands when Ru is substituted for Co in Co_2−*x*_Ru_*x*_MnSi compounds. When Ru content is higher than 0.7 (*x* > 0.7), the Fermi level crosses through the top of the lower-value band and the spin-flip band gap no longer exists. It should be noted that, although the Co_2−*x*_Ru_*x*_MnSi compounds are no longer rigorous HM materials when *x* > 0.7, they still have a very high spin-polarization of conduction electrons which can be confirmed from the DOS patterns shown in Fig. 3 ▸. Furthermore, for Co_2−*x*_Ru_*x*_MnSi (*x* > 0.7) compounds, it can be pointed out that the electron carriers are in 100% spin-polarization based on the band structure features near the Fermi level.

Fig. 3 ▸ shows the spin-projected TDOS (total density of states) and PDOS (partial density of states) patterns for the Co_2−*x*_Ru_*x*_MnSi (*x* = 0, 0.25, 0.5, 0.75, 1) compounds. Fig. 3 ▸ shows that the DOS on both edges of the band gap in the spin-down channel is mainly composed of the hybridized 3*d* states of Co(A) and Co(C)/Ru(C) atoms, and the Mn(B) atom has a larger band gap containing the band gaps of Co(A) and Co(C)/Ru(C) atoms. This suggests that the band gap is determined by the Co(A) and Co(C)/Ru(C) atoms. In association with the band structures in Fig. 1 ▸, we can identify that the states on both edges of the band gap are the doubly degenerate *e*_u_ states and triply degenerate *t*_1u_ states, which only originate from the hybridization with Co(A) and Co(C)/Ru(C) *d* states. The *e*_u_ and *t*_1u_ orbitals cannot couple with any of the Mn(B) *d* orbitals as these are not transformed with the u representations (Galanakis *et al.*, 2002 ▸; Liu *et al.*, 2008 ▸). Therefore, it is clear that the band gap of the Co(A) and Co(C)/Ru(C) atoms is the *d*–*d* band gap arising only from the *e*_u_–*t*_1u_ splitting generated mainly by the crystal field, which is similar to the reported Co_2_MnZ and Mn_2_CoZ alloys with the stoichiometric ratio of Heusler compounds (Galanakis *et al.*, 2002 ▸; Liu *et al.*, 2008 ▸). The exchange splitting will shift the Fermi level to the appropriate position. Comparing the PDOS pattern of a Co atom with that of Ru shows that Ru has a smaller spin-splitting than Co and hardly affects the band gap. Since Ru has one less valence electron than Co, the Fermi level has to shift to a lower energy with increasing Ru content, which leads to the continuous change of the spin-flip band gap with Ru content. This indicates that Co_2−*x*_Ru_*x*_MnSi (*x* = 0, 0.25, 0.5, 0.75, 1) compounds are a series of HM materials with adjustable spin-flip band gaps.

### 3.2. Preparation and crystal structure   

**Figure 4:**
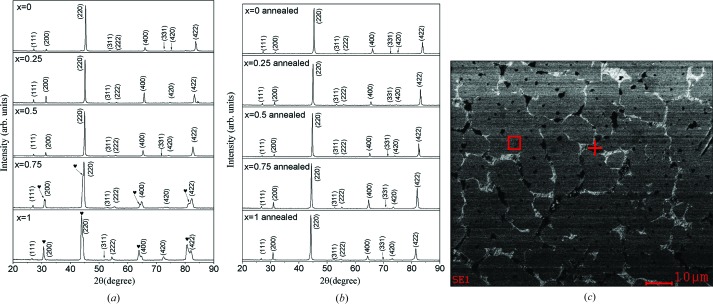
(*a*) PXRD patterns of arc-melt Co_2−*x*_Ru_*x*_MnSi (*x* = 0, 0.25, 0.50, 0.75, 1.00) ingots. (*b*) PXRD patterns of arc-melt Co_2−*x*_Ru_*x*_MnSi (*x* = 0, 0.25, 0.50, 0.75 and 1.00) ingots annealed at 1173 K for 2 days. (*c*) SEM image of the metallographic specimen of the arc-melt Co_1.25_Ru_0.75_MnSi.

Room-temperature PXRD patterns of arc-melt Co_2−*x*_Ru_*x*_MnSi (*x* = 0, 0.25, 0.5, 0.75, 1) ingots are shown in Fig. 4 ▸(*a*). The diffraction peaks are indexed and the corresponding Miller indices are also tagged in the patterns. In Fig. 4 ▸(*a*), it can be seen that not all the Co_2−*x*_Ru_*x*_MnSi (*x* = 0, 0.25, 0.50, 0.75, 1) compounds can be synthesized as single phase only by using the arc-melt method. For the Co_2−*x*_Ru_*x*_MnSi (*x* = 0, 0.25) compositions, all the principal reflection peaks of a body-centered cubic (b.c.c.) structure are observed without the other secondary peaks in the PXRD patterns, indicating that the Co_2−*x*_Ru_*x*_MnSi (*x* = 0, 0.25) compounds form the pure b.c.c. structure. However, for the Co_1.5_Ru_0.5_MnSi, Co_1.25_Ru_0.75_MnSi and CoRuMnSi compositions, it is clear that there are two sets of reflection peaks of a b.c.c. structure in the PXRD patterns, which indicates that two phases were formed in the arc-melt Co_2−*x*_Ru_*x*_MnSi (*x* = 0.5, 0.75, 1) ingots. As a typical representative composition, the metallographic specimen of the arc-melt Co_1.25_Ru_0.75_MnSi was observed by SEM and the images are shown in Fig. 4 ▸(*c*). It is very clear that there are two different areas. In the images, the red box and red cross are the measuring points of the energy-dispersive X-ray spectroscopy (EDS), respectively. By EDS, the Co_1.7_Ru_0.3_MnSi and Ru_2_MnSi compositions can be detected in the arc-melt Co_1.25_Ru_0.75_MnSi composition. In association with the PXRD patterns, we can determine that two phases with Co_1.7_Ru_0.3_MnSi and Ru_2_MnSi compositions and b.c.c. structures are formed in the arc-melt Co_2−*x*_Ru_*x*_MnSi (*x* = 0.5, 0.75, 1) ingots.

In order to obtain a single phase of the Co_2−*x*_Ru_*x*_MnSi (*x* = 0.5, 0.75, 1) compositions, annealing experiments at various temperatures were carried out. It was found that a heat-treatment process of 1173 K for 48 h is suitable to form a single phase with a b.c.c. structure for the Co_2−*x*_Ru_*x*_MnSi (*x* = 0.5, 0.75, 1) compositions. The PXRD patterns are shown in Fig. 4 ▸(*b*) for the arc-melt Co_2−*x*_Ru_*x*_MnSi (*x* = 0, 0.25, 0.5, 0.75, 1) ingots annealed at 1173 K for 48 h. It can be seen that all the annealed Co_2−*x*_Ru_*x*_MnSi (*x* = 0, 0.25, 0.5, 0.75, 1) compositions have a single phase with a b.c.c. structure. Furthermore, it was known that for Heusler compounds, the (111) and (200) diffraction peaks correspond to the order-dependent superlattice reflections (Bacon & Plant, 1971 ▸). It can be found that the (111) and (200) superlattice reflection peaks occur on all the PXRD patterns of the arc-melt Co_2−*x*_Ru_*x*_MnSi (*x* = 0, 0.25, 0.5, 0.75, 1) ingots annealed at 1173 K for 48 h, which strongly suggests that a highly ordered *L*2_1_ structure has been formed in these ingots. A highly ordered crystal structure is necessary to obtain high spin polarization in these materials (Felser *et al.*, 2007 ▸). However, it should be noted that for the Co_2_MnSi compound (namely, the case of *x* = 0), the result is consistent with early reports (Rabie *et al.*, 2017 ▸).

**Figure 5:**
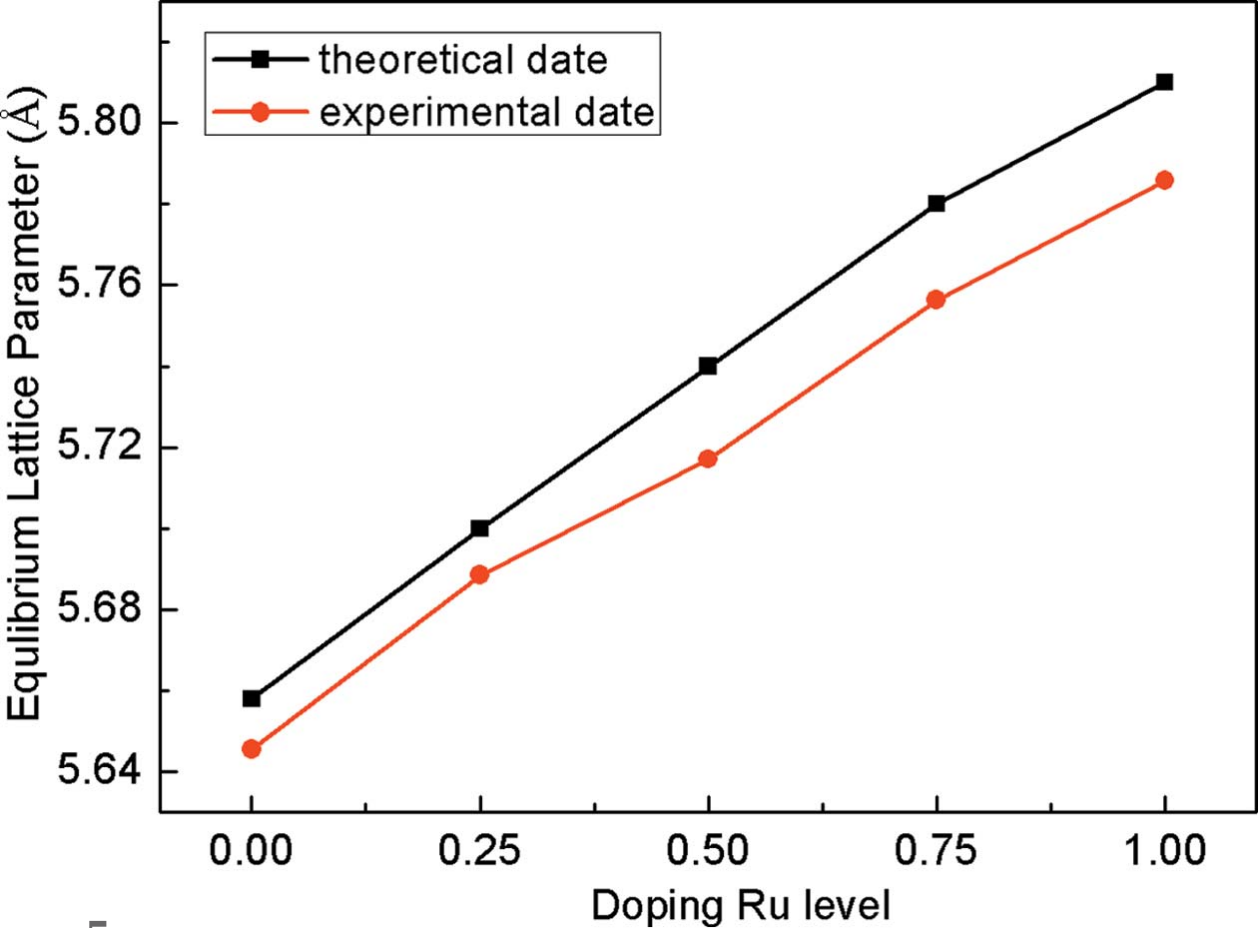
Ru-level dependence of the ELP in theory (black dot + line) and the experimental lattice parameter (red dot + line).

The Rietveld refinement was used to fit the experimental PXRD data for the lattice parameters. The experimental lattice parameters are gathered in Table 1 ▸ and plotted as a function of Ru content in Fig. 5 ▸. The Ru-content dependence of ELP in theory is also shown in Fig. 5 ▸ for the Co_2−*x*_Ru_*x*_MnSi (*x* = 0, 0.25, 0.5, 0.75, 1) compounds. It should be pointed out that the theoretical data values are slightly larger than the experimental results. This phenomenon is observed in many Heusler compounds and can be attributed to the overestimation of lattice parameters by GGA in first-principles calculations (Picozzi & Freeman, 2015 ▸; Aguayo & Murrieta, 2011 ▸; Page *et al.*, 2015 ▸). In Fig. 5 ▸, it can be seen that the lattice parameter increases with the increase in Ru content due to the larger atomic radius of Ru than Co. The range of experimental lattice parameters is between 5.646 and 5.786 Å for the Co_2−*x*_Ru_*x*_MnSi (*x* = 0, 0.25, 0.5, 0.75, 1) compounds, indicating that the lattice parameter can be tunable up to 2.5% by Ru-doping.

### 3.3. Magnetic properties   

**Figure 6:**
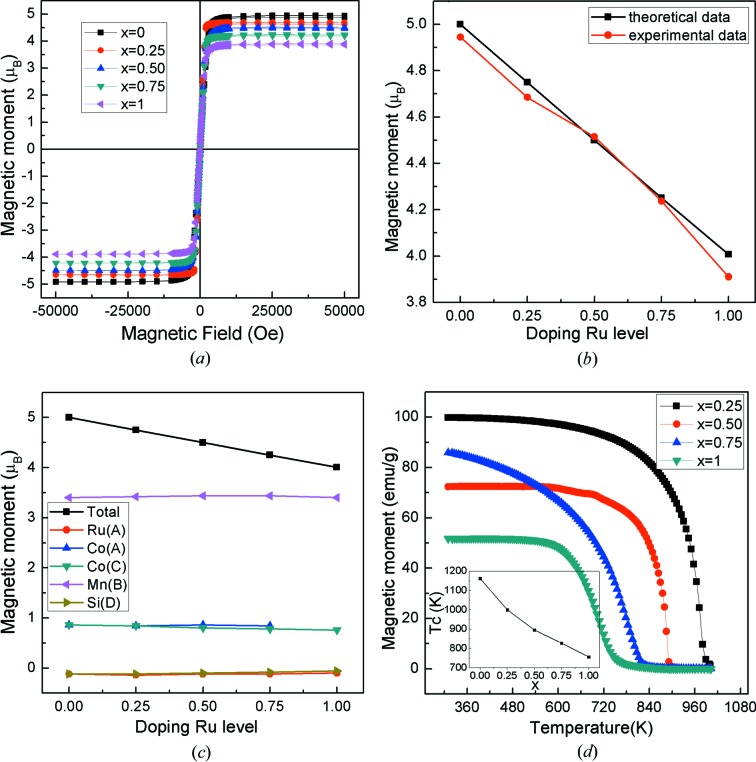
(*a*) Magnetization curves (M–H curves) of Co_2−*x*_Ru_*x*_MnSi (*x* = 0, 0.25, 0.5, 0.75, 1) alloys recorded at 3 K in the field range ±5 T. (*b*) Experimental and theoretical magnetization as a function of the Ru-doping level. (*c*) Calculated atomic magnetic moments and total magnetic moments as a function of the Ru-doping level. (*d*) Magnetization curves (M–T curves) of Co_2−*x*_Ru_*x*_MnSi (*x* = 0.25, 0.5, 0.75, 1) alloys at 310–1000 K.

From the TDOS (in Fig. 3 ▸) and band structures (in Fig. 1 ▸), it is clear that the *e*_u_ states are above the Fermi level, whereas the *t*_1u_ states are just below the Fermi level in the spin-down channel. So, the total eight minority *d* bands are filled in the spin-down channel for the Co_2−*x*_Ru_*x*_MnSi (*x* = 0, 0.25, 0.5, 0.75, 1) compounds. According to the explanations of Galanakis *et al.* on the origin of the band gap and Slater–Pauling rule in HM Heusler compounds (Galanakis *et al.*, 2002 ▸), the Co_2−*x*_Ru_*x*_MnSi (x = 0, 0.25, 0.5, 0.75, 1) compounds should follow the Slater–Pauling rule: *M*_t_ = *Z*_t_ − 24 (*M*_t_ and *Z*_t_ are the spin magnetic moment and the number of valence electrons per unit cell). The magnetization curves were experimentally detected and shown in Fig. 6 ▸(*a*) for the Co_2−*x*_Ru_*x*_MnSi (*x* = 0, 0.25, 0.5, 0.75, 1) compounds. In Fig. 6 ▸(*a*), it shows that the Co_2−*x*_Ru_*x*_MnSi (*x* = 0, 0.25, 0.5, 0.75, 1) compounds exhibit ferromagnetic behavior with a small coercive field. The calculated total magnetic moments and experimental saturation magnetization dependence on Ru content are plotted in Fig. 6 ▸(*b*). It is clear that the experimental results are in good agreement with the theoretical results, with an error of less than 1%. The total magnetic moment (per unit cell) follows the *M*_t_ = *Z*_t_ − 24 Slater–Pauling rule and changes continuously from 5 to 4 μ_B_ on increasing Ru content from *x* = 0 to 1. We know that the greater the spin-splitting between spin-up and spin-down states, the larger localized spin magnetic moments. From the PDOS patterns shown in Fig. 3 ▸, it can be seen that Mn and Co atoms have a strong spin-splitting and the spin-splitting of Ru is small and almost negligible, which indicates that Mn and Co atoms are the major contributors to the total magnetic moment in Co_2−*x*_Ru_*x*_MnSi compounds. In order to illustrate the magnetic structure and how the atomic magnetic moments change with the doping Ru content, the calculated atomic magnetic moments based on the PDOS were gathered and plotted in Fig. 6 ▸(*c*) for the Co_2−*x*_Ru_*x*_MnSi (*x* = 0, 0.25, 0.5, 0.75, 1) compounds. It is clear that the Co atoms have a magnetic moment of 0.86 μ_B_ and the Mn atoms 3.4 μ_B_. The magnetic moments of Co and Mn atoms are parallel and remain stable throughout the whole composition range from Co_2_MnSi to CoRuMnSi. Therefore, the decrease of the total magnetic moment with the increase in Ru content can only be attributed to the decrease in the number of Co atoms and the doped Ru atoms have no effect on the magnetic moment of the other atoms.

The Curie temperature is an important parameter for the application of HM materials. A high Curie temperature usually implies stable magnetism and half-metallicity in a wide temperature range, which is conductive to practical applications. Here, Curie temperatures were measured using M–T curves and plotted as a function of Ru content in Fig. 6 ▸(*d*). For the Co_2_MnSi compound, our result is consistent with that previously reported by Akriche *et al.*, (2017 ▸). In Fig. 6 ▸(*d*), it can be seen that the Curie temperature monotonously decreases with increasing Ru content, which can be attributed to the weakening of the exchange interaction caused by the doped Ru atoms with a small magnetic moment. Although the Curie temperature decreases with increasing Ru content, it remains higher than 750 K for all the Co_2−*x*_Ru_*x*_MnSi (*x* = 0, 0.25, 0.5, 0.75, 1) compounds. Co_2−*x*_Ru_*x*_MnSi (*x* = 0, 0.25, 0.5, 0.75, 1) compounds are a rare case of HM materials with such high Curie temperatures.

### 3.4. Electronic transport properties   

**Figure 7:**
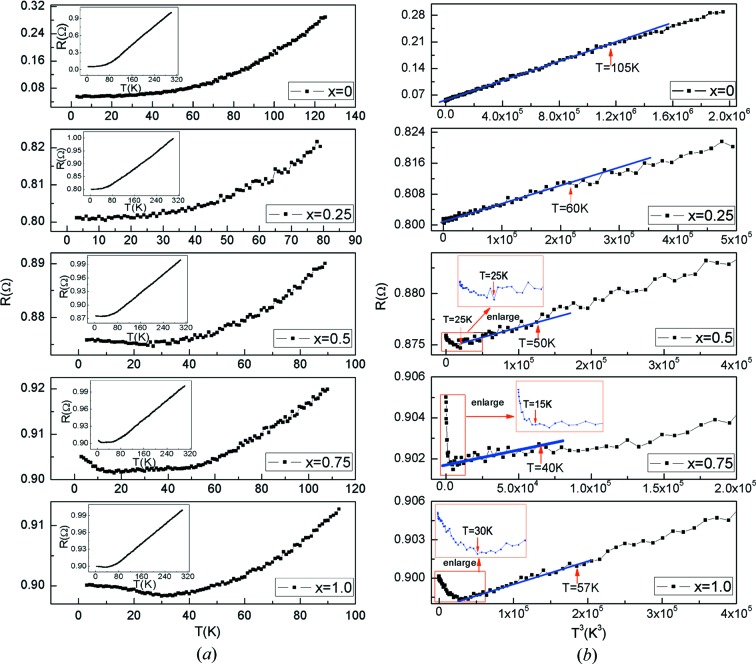
(*a*) Local magnification of the *T*-plot at low temperature with inserts that are the curves of resistance with temperature for Co_2−*x*_Ru_*x*_MnSi (*x* = 0, 0.25, 0.5, 0.75, 1) compounds. (*b*) *T* ^3^-plot of the resistance *R*(*T*) at low temperature for all the Co_2−*x*_Ru_*x*_MnSi (*x* = 0, 0.25, 0.5, 0.75, 1) compounds.

It has been reported that the resistance has a *T* ^3^ (where *T* is temperature) instead of *T* ^2^ dependence for common metals at low temperature for HM materials (Furukawa, 2000 ▸). The *T* ^3^ dependence of resistance is a unique characteristic of HM materials (Furukawa, 2000 ▸). Fig. 7 ▸(*a*) shows that the curves of resistance dependence on temperature for the Co_2−*x*_Ru_*x*_MnSi (*x* = 0, 0.25, 0.5, 0.75, 1) compounds. It can be observed that the resistance linearly increases with increasing temperature in the high-temperature range for all Co_2−*x*_Ru_*x*_MnSi (*x* = 0, 0.25, 0.5, 0.75, 1) compounds (note: compounds with different compositions have different temperature ranges). The linear dependence of resistance with temperature is similar to the reported HM Heusler compound Co_2_MnSi (Raphael *et al.*, 2002 ▸). However, in the low-temperature range, the dependence of the resistance on temperature deviates from linear behavior. In order to understand the low-temperature behavior of resistance, we use 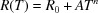 (where *R* is the resistance, *R*_0_ is the residual resistance, *T* is temperature, *A* is a coefficient and *n* is the power exponent) to fit the experimental data. Fig. 7 ▸(*b*) shows the *T* ^3^-plot of the resistance *R*(*T*) and local magnification of the *T*-plot at low temperature for all of the Co_2−*x*_Ru_x_MnSi (*x* = 0, 0.25, 0.5, 0.75, 1) compounds. In Fig. 7 ▸(*b*), it can be clearly observed that the resistance data fit well in the form 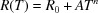 with *n* = 3 in the low-temperature region for Co_2_MnSi and Co_1.75_Ru_0.25_MnSi. Nobuo Furukawa pointed out that *T* ^3^ resistance can be used as a probe to investigate whether a given compound is a half-metal (Furukawa, 2000 ▸). The *T* ^3^ resistance of Co_2_MnSi and Co_1.75_Ru_0.25_MnSi indicates well that these two compounds are HM, which is also consistent with the prediction based on their band structures as illustrated in Section 3.1.

Furthermore, the semiconductive/half-metallic resistance characteristics were observed in the temperature range below 25, 15 and 30 K for the Co_2−*x*_Ru_*x*_MnSi (*x* = 0.5, 0.75, 1) compounds with high Ru content. In other words, the resistance decreases with increasing temperature in these compounds when the temperature is lower than 25, 15 and 30 K. The phenomenon can be interpreted with the band structures shown in Figs. 1 ▸(*c*), 1(*d*) and 1(*e*) again. As illustrated in Section 3.1, the compounds are not rigorous half-metals when the content of Ru is higher than 0.7 (*x* > 0.7) because their Fermi level touches or slightly crosses through the top of valence bands at the G point in the spin-down channel. Such a band structure in the spin-down channel is typical for a half-metal. For a half-metal, a large number of carriers are excited with increasing temperature, which leads to a decrease in resistance. Obviously, in the spin-up channel, a typical metallic band structure was observed. This means a competition of resistance behavior in two spin directions occurs in the Co_2−*x*_Ru_*x*_MnSi (*x* = 0.75, 1) compounds, which leads to the final temperature dependence of resistance.

## 4. Conclusions   

We have successfully synthesized a series of Co_2−*x*_Ru_*x*_MnSi (*x* = 0, 0.25, 0.5, 0.75, 1) Heusler compounds by annealing the samples at 1173 K for 48 h. The electronic structures achieved by first-principles calculations indicate that the Co_2−*x*_Ru_*x*_MnSi (*x* < 0.7) compounds are rigorous half-metals and the Co_2−*x*_Ru_*x*_MnSi (*x* ≥ 0.7) compounds are nearly half-metals. Structural measurements show that the lattice parameters change from 5.646 to 5.786 Å on increasing the Ru-doping level. The magnetic moments follow the Slater–Pauling rule and can be continuously tuned in the range 4–5 μ_B_. The doped Ru atom does not affect the band gap but leads to the continuous change of the spin-flipping band gap. The resistance shows a *T* ^3^ dependence behavior which further indicates the half-metallicity of Co_2−*x*_Ru_*x*_MnSi (*x* = 0, 0.25). The Curie temperatures of all the Co_2−*x*_Ru_*x*_MnSi (*x* = 0, 0.25, 0.5, 0.75, 1) Heusler compounds are above 750 K. The results reveal that the Co_2−*x*_Ru_*x*_MnSi (*x* = 0, 0.25, 0.5, 0.75, 1) Heusler compounds are a series of real highly spin-polarized materials and good candidates for spintronics applications.

## Figures and Tables

**Table 1:** The equilibrium lattice parameters, total and partial spin moments of the Co_2−*x*_Ru_*x*_MnSi (*x* = 0, 0.25, 0.5, 0.75, 1) series alloys obtained by theoretical calculation and geometry optimization

Ru content (*x*)	ELP (Å)	*M*_t_ (μ_B_)	*M*_Co(C)_ (μ_B_)	*M*_Co(A)_ (μ_B_)	*M*_Ru(C)_ (μ_B_)	*M*_Mn(B)_ (μ_B_)	*M*_Si(D)_ (μ_B_)
0	5.66	5.00	0.86	0.86	–	3.40	−0.12
0.25	5.70	4.75	0.84	0.84	−0.14	3.42	−0.12
0.50	5.74	4.50	0.86	0.80	−0.12	3.44	−0.10
0.75	5.78	4.25	0.84	0.78	−0.12	3.44	−0.08
1.0	5.81	4.01	–	0.76	−0.10	3.40	−0.06
